# Supplementary material for: Determination of CYP450 Expression Levels in the Human Small Intestine by Mass Spectrometry-Based Targeted Proteomics
Source: Int J Mol Sci. 2021 Nov 26;22(23):12791. doi: 10.3390/ijms222312791 (PMC8657875; doi:10.3390/ijms222312791)
Supplement: Supplementary file 1 [file ijms-22-12791-s001.zip › ijms-1414658-supplementary.pdf]

Supplemental data

Figure S1. Representative chromatograms of the overlay of intestinal microsomes from one tissue donor, a blank with internal standard and a standard at 2 nM.

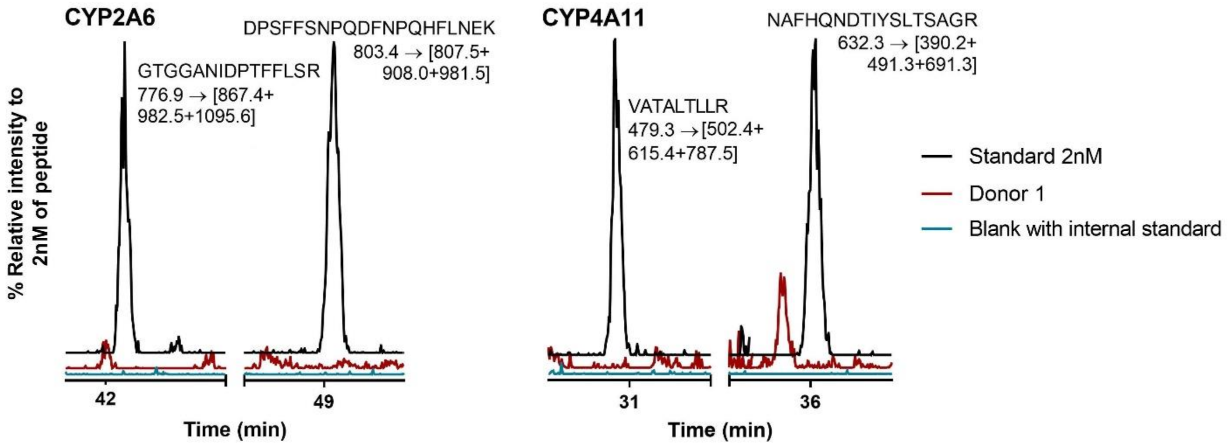

Table S1. Proteospecific peptides sequences, MRM transitions (precursor to products transitions), and collision energy (CE) used for quantification. The labeling of lysine or arginine terminal is indicated in the internal standard sequence by K^ or R^.

Protein	Proteotypic peptide	MWs	Precursor (m/z)	z	Product (m/z)	Ion/z	CE (eV)	Product (m/z)	Ion/z	CE (eV)	Product (m/z)	Ion/z	CE (eV)
CYP2A6	GTGGANIDPTFFLSR	1552.7	776.9	2	867.4	y7/1+	31.5	982.5	y8/1+	26.8	1095.6	y9/1+	27.4
	GTGGANIDPTFFLSR^	1562.6	781.9	2	877.4	y7/1+	31.5	992.5	y8/1+	26.8	1105.6	y9/1+	27.4
	DPSFFSNPQDFNPQHFLNEK	2408.6	803.4	3	807.5	y13/2+	26.1	908.0	y15/2+	23.9	981.5	y16/2+	22.7
	DPSFFSNPQDFNPQHFLNEK^	2416.5	806.0	3	811.5	y13/2+	26.1	912.1	y15/2+	23.9	985.5	y16/2+	22.7
CYP4A11	VATALTLLR	957.2	479.3	2	502.4	y4/1+	17.2	615.4	y5/1+	18.5	787.5	y7/1+	17.4
	VATALTLLR^	967.1	484.3	2	512.4	y4/1+	17.2	625.4	y5/1+	18.5	797.5	y7/1+	17.4
	NAFHQNDTIYSLTSAGR	1895.0	632.3	3	390.2	y4/1+	16.2	491.3	y5/1+	17.0	691.3	y7/1+	20.6
	NAFHQNDTIYSLTSAGR^	1904.9	635.6	3	400.2	y4/1+	16.2	501.3	y5/1+	17.0	701.3	y7/1+	20.6

Table S2. Summary of intra-day (six replicates per concentration) and inter-day (three individual runs) precision and accuracy of quality control samples for proteotypic peptides in ammonium bicarbonate buffer (NH_4HCO_3). Results are expressed as concentration (mean ± SD); RSD, relative standard deviation; RE, relative error.

**Protein**	**Conc. (nM)**	**Peptide**	**Intra-day (n=6)**			**Inter-day (n=18)**		
			**Mean ± SD (nM)**	**RSD (%)**	**RE (%)**	**Mean ± SD (nM)**	**RSD (%)**	**RE (%)**
CYP2A6	0.1	GTGGANIDPTFFLSR	0.095 ± 0.013	13.6	-5.5	0.11 ± 0.019	17.2	8.5
	0.5		0.52 ± 0.043	8.3	4.7	0.53 ± 0.042	8.0	6.1
	5		4.7 ± 0.17	3.7	-6.0	5.0 ± 0.30	5.9	-0,07
	15		14.8 ± 0.896	6.0	-1.2	14.7 ± 0.689	4.7	-2.1
CYP2A6	0.1	DPSFFSNPQDFNPQHFLNEK	0.11 ± 0.01	11.1	14.3	0.10 ± 0.016	15.1	3.6
	0.5		0.54 ± 0.03	6.3	7.6	0.53 ± 0.036	6.7	6.7
	5		4.8 ± 0.29	6.1	-4.7	5.1 ± 0.30	5.9	1.6
	15		15.5 ± 0.82	5.3	3.5	15.1 ± 0.66	4.4	0.54
CYP4A11	0.1	NAFHQNDTIYSLTSAGR	0.081 ± 0.014	17.4	-19.3	0.088 ± 0.017	19.8	-11.6
	0.5		0.52 ± 0.034	6.6	3.7	0.49 ± 0.053	10.7	-1.7
	5		5.0 ± 0.19	3.7	0.70	5.2 ± 0.30	5.7	5.0
	15		14.6 ± 0.695	4.7	-2.4	15.6 ± 1.5	9.5	3.8
CYP4A11	0.1	VATALTLLR	0.09 ± 0.18	19.3	-7.8	0.096 ± 0.019	19.8	-4.0
	0.5		0.53 ± 0.031	5.8	5.0	0.51 ± 0.048	9.4	1.5
	5		5.0 ± 0.091	1.8	0.16	5.0 ± 0.23	4.6	0.70
	15		14.9 ± 0.66	4.4	-0.53	14.7 ± 1.06	7.2	-2.2

Table S3. Summary of matrix effect in self-prepared HIM for all proteotypic peptides. Results are expressed as percent nominal ± SD, n=3.

Protein	Peptide	Concentration (nM)	%Nominal (mean ± SD, n=3) Matrice 1	Matrice 2	Matrice 3	Matrice 4	Matrice 5	Matrice 6
CYP2A6	GTGGANIDPTFFLSR	5	102.8 ± 1.5	90.0 ± 14.1	98.5 ± 3.6	110.1 ± 4.1	119.2 ± 16.7	111.3 ± 5.4
		15	94.3 ± 7.7	92.2 ± 2.9	86.1 ± 0.8	80.7 ± 6.7	90.4 ± 7.8	97.5 ± 2.1
	DPSFFSNPQDFNPQHFLNEK	5	92.5 ± 9.7	80.4 ± 6.2	88.1 ± 5.0	110.4 ± 1.8	89.1 ± 8.8	94.6 ± 12.7
		15	85.2 ± 6.6	85.8 ± 11.1	86.0 ± 9.6	90.6 ± 10.8	97.1 ± 4.2	86.1 ± 3.2
CYP4A11	NAFHQNDTIYSLTSAGR	5	102.1 ± 6.8	90.9 ± 7.1	86.1 ± 3.6	80.3 ± 9.7	90.4 ± 5.3	96.8 ± 6.1
		15	90.9 ± 5.5	87.2 ± 4.5	100.6 ± 7.5	81.2 ± 3.3	85.3 ± 2.6	92.0 ± 5.0
	VATALTLLR	5	83.9 ± 11.5	98.7 ± 13.8	106.3 ± 9.9	89.7 ± 16.4	99.9 ± 11.7	104.1 ± 17.2
		15	87.1 ± 1.5	108.7 ± 7.3	107.5 ± 6.3	109.5 ± 5.2	100.2 ± 3.6	108.6 ± 11.5

Table S4. Autosampler stability results for proteotypic peptides. Results are expressed as percent nominal ± SD, n=6.

Protein	Peptide	Conc. (nM)	Autosampler (98h, 15˚C), n=6 %Nominal (mean ± SD)	Peptide	Conc. (nM)	Autosampler (98h, 15˚C), n=6 %Nominal (mean ± SD)
CYP2A6	GTGGANIDPTFFLSR	0.5	103.4 ± 3.9	DPSFFSNPQDFNPQHFLNEK	0.5	108.5 ± 2.1
		5	103.4 ± 2.9		5	96.3 ± 0.6
		15	98.8 ± 8.4		15	99.2 ± 9.2
CYP4A11	NAFHQNDTIYSLTSAGR	0.5	109.2 ± 3.1	VATALTLLR	0.5	106.9 ± 10
		5	98.0 ± 6.2		5	95.4 ± .5
		15	93.9 ± 3.0		15	88.0 ± 3.7

Table S5. Correlation between proteotypic peptides using the Spearman rank test for each CYP450 isoforms.

Protein	n	r (Spearman)	Y-intercepts
CYP2A6	16	0.694*	-2.764
CYP4A11	16	0.679†	4.099

r, correlation coefficient

**p*≤0.05, †*p*≤0.001

Table S6. Protein quantification in commercially available pooled HLM, HIM and self-prepared HIM. Protein amounts are expressed as mean ± SD, n=3.

Protein	Protein amount (pmol/mg of protein, mean ± SD)					
	Commercial pooled HLM 1	Commercial pooled HLM 2	Commercial pooled HIM	Med. Jejunum donor 1	Med. Jejunum donor 2	Med. Jejunum don
CYP2A6	18,6 ± 16,4	23,7 ± 22,5	ND	ND	ND	ND
CYP4A11	4,4 ± 2,0	4,1 ± 2,1	ND	ND	ND	ND

Table S7. Protein expression of 16 CYP450 isoenzymes for each donor in 7 intestinal subsections. Protein amounts are expressed are expression in pmol/mg prot.

**Protein**	**IN001**													
	**Duodenum**		**Proximal jejunum**		**Mid-jejunum**		**Distal jejunum**		**Proximal ileum**		**Mid-ileum**		**Distal ileum**	
	**Mean**	**SD**	**Mean**	**SD**	**Mean**	**SD**	**Mean**	**SD**	**Mean**	**SD**	**Mean**	**SD**	**Mean**	**SD**
CYP1A1	ND		ND		ND		ND		ND		ND		ND	
CYP1A2	ND		ND		ND		ND		ND		ND		ND	
CYP1B1	ND		ND		ND		ND		ND		ND		ND	
CYP2A6	ND		ND		ND		ND		ND		ND		ND	
CYP2B6	ND		ND		ND		ND		ND		ND		ND	
CYP2C8	ND		ND		ND		ND		ND		ND		ND	
CYP2C9	2.16	0.59	3.83	0.51	3.8	1.5	0.97	0.29	0.78	0.77	0.44	0.27	0.0	
CYP2C19	0.87	0.25	0.62	0.14	0.37	0.40	0.10	0.11	0.15	0.15	0.21	0.15	0.05	0.06
CYP2D6	0.03	0.03	0.05	0.03	0.14	0.09	0.05	0.05	0.04	0.04	0.14	0.12	0.02	0.04
CYP2E1	ND		ND		ND		ND		ND		ND		ND	
CYP2J2	0.16	0.16	0.36	0.31	0.63	0.52	0.52	0.32	0.51	0.46	0.69	0.48	0.30	0.23
CYP3A4	12	1.5	16	1.7	24	3.1	6.8	3.3	15	4.6	15	1.1	5.3	0.93
CYP3A5	0.28	0.08	0.49	0.08	0.89	0.15	0.64	0.29	0.76	0.39	1.1	0.38	0.64	0.19
CYP3A7	ND		ND		ND		ND		ND		ND		ND	
CYP4A11	ND		ND		ND		ND		ND		ND		ND	
CYP4F2	1.47	0.12	3.2	0.24	4.9	0.46	2.1	0.37	3.9	0.87	3.9	0.20	2.1	0.22

**Protein**	**IN002**													
	**Duodenum**		**Proximal jejunum**		**Mid-jejunum**		**Distal jejunum**		**Proximal ileum**		**Mid-ileum**		**Distal ileum**	

	Mean	SD	Mean	SD	Mean	SD	Mean	SD	Mean	SD	Mean	SD	Mean	SD
CYP1A1	NA		ND		ND		ND		ND		ND		ND	
CYP1A2	NA		ND		ND		ND		ND		ND		ND	
CYP1B1	NA		ND		ND		ND		ND		ND		ND	
CYP2A6	NA		ND		ND		ND		ND		ND		ND	
CYP2B6	NA		ND		ND		ND		ND		ND		ND	
CYP2C8	NA		ND		ND		ND		ND		ND		ND	
CYP2C9	NA		11	1.0	9.9	1.7	4.7	0.86	2.9	1.3	3.3	0.90	2.0	1.1
CYP2C19	NA		3.0	0.43	2.0	0.28	0.60	0.07	0.44	0.20	0.62	0.20	0.40	0.18
CYP2D6	NA		0.76	0.06	0.87	0.18	0.46	0.16	0.15	0.09	0.29	0.06	0.35	0.14
CYP2E1	NA		ND		ND		ND		ND		ND		ND	
CYP2J2	NA		0.63	0.31	0.76	0.34	0.65	0.35	0.54	0.30	1.0	0.23	0.76	0.09
CYP3A4	NA		55	4.5	58	3.8	32	4.2	39	6.6	28	1.9	45	5.5
CYP3A5	NA		0.95	0.11	0.93	0.22	0.52	0.13	0.49	0.32	0.80	0.37	0.72	0.60
CYP3A7	NA		ND		ND		ND		ND		ND		ND	
CYP4A11	NA		ND		ND		ND		ND		ND		ND	
CYP4F2	NA		5.8	0.36	8.1	0.55	5.1	0.43	5.3	0.69	5.0	0.15	4.0	0.53

Protein	IN003						
	Duodenum	Proximal jejunum	Mid-jejunum	Distal jejunum	Proximal ileum	Mid-ileum	Distal ileum

	Mean	SD	Mean	SD	Mean	SD	Mean	SD	Mean	SD	Mean	SD	Mean	SD
CYP1A1	ND		ND		ND		ND		ND		ND		ND	
CYP1A2	ND		ND		ND		ND		ND		ND		ND	
CYP1B1	ND		ND		ND		ND		ND		ND		ND	
CYP2A6	ND		ND		ND		ND		ND		ND		ND	
CYP2B6	ND		ND		ND		ND		ND		ND		ND	
CYP2C8	ND		ND		ND		ND		ND		ND		ND	
CYP2C9	1.9	1.5	3.7	2.4	3.2	2.2	3.6	2.8	2.1	1.7	3.5	1.3	2.0	1.4
CYP2C19	0.55	0.21	0.58	0.23	0.35	0.19	0.22	0.20	0.19	0.18	0.26	0.16	0.42	0.19
CYP2D6	0.15	0.13	1.0	0.32	0.78	0.33	0.57	0.28	0.37	0.29	1.6	0.37	1.5	1.3
CYP2E1	ND		ND		ND		ND		ND		ND		ND	
CYP2J2	0.17	0.15	0.52	0.33	0.55	0.41	0.86	0.80	0.79	0.65	1.1	0.37	0.56	0.35
CYP3A4	25	11	40	14	46	20	57	35	39	31	36	7.2	29	16
CYP3A5	0.19	0.16	0.38	0.13	0.35	0.15	0.64	0.47	0.48	0.40	0.60	0.30	0.50	0.40
CYP3A7	ND		ND		ND		ND		ND		ND		ND	
CYP4A11	ND		ND		ND		ND		ND		ND		ND	
CYP4F2	2.7	0.38	8.3	0.84	9.6	0.32	13	2.7	8.6	0.74	9.3	0.54	8.4	0.30

**Protein**	**IN004**						
	**Duodenum**	**Proximal jejunum**	**Mid-jejunum**	**Distal jejunum**	**Proximal ileum**	**Mid-ileum**	**Distal ileum**

	Mean	SD	Mean	SD	Mean	SD	Mean	SD	Mean	SD	Mean	SD	Mean	SD
CYP1A1	ND		ND		ND		ND		ND		ND		ND	
CYP1A2	ND		ND		ND		ND		ND		ND		ND	
CYP1B1	ND		ND		ND		ND		ND		ND		ND	
CYP2A6	ND		ND		ND		ND		ND		ND		ND	
CYP2B6	ND		ND		ND		ND		ND		ND		ND	
CYP2C8	ND		ND		ND		ND		ND		ND		ND	
CYP2C9	7.8	2.5	7.8	3.4	6.2	1.6	4.4	2.1	1.6	1.4	1.9	0.59	1.4	0.66
CYP2C19	2.4	0.75	2.1	0.45	1.9	0.53	0.84	0.46	0.45	0.23	0.40	0.23	0.51	0.19
CYP2D6	0.0		0.02	0.02	0.03	0.06	0.0		0.0		0.0		0.0	
CYP2E1	ND		ND		ND		ND		ND		ND		ND	
CYP2J2	1.1	0.70	1.5	0.68	1.8	0.74	2.0	0.93	1.9	1.1	2.0	0.56	1.7	0.75
CYP3A4	46	6.7	36	6.4	25	1.7	41	6.4	23	12	25	3.4	16	6.7
CYP3A5	0.74	0.34	0.71	0.31	0.67	0.19	0.62	0.29	0.73	0.55	0.75	0.43	0.64	0.31
CYP3A7	ND		ND		ND		ND		ND		ND		ND	
CYP4A11	ND		ND		ND		ND		ND		ND		ND	
CYP4F2	2.0	0.21	2.8	0.39	2.5	0.27	4.0	0.44	3.3	0.66	1.5	0.24	1.5	0.16

**Protein**	**IN005**						
	**Duodenum**	**Proximal**	**Mid-**	**Distal jejunum**	**Proximal ileum**	**Mid-ileum**	**Distal ileum**

			jejunum		jejunum									
	**Mean**	**SD**	**Mean**	**SD**	**Mean**	**SD**	**Mean**	**SD**	**Mean**	**SD**	**Mean**	**SD**	**Mean**	**SD**
CYP1A1	ND		ND		ND		ND		ND		ND		ND	
CYP1A2	ND		ND		ND		ND		ND		ND		ND	
CYP1B1	ND		ND		ND		ND		ND		ND		ND	
CYP2A6	ND		ND		ND		ND		ND		ND		ND	
CYP2B6	ND		ND		ND		ND		ND		ND		ND	
CYP2C8	ND		ND		ND		ND		ND		ND		ND	
CYP2C9	5.9	2.4	6.9	3.1	5.6	0.88	3.7	18	3.8	1.4	1.6	1.0	1.4	0.83
CYP2C19	1.9	0.41	2.1	0.99	2.0	0.28	0.57	0.23	0.81	0.23	0.43	0.21	0.40	0.15
CYP2D6	0.96	0.12	1.3	0.33	1.1	0.09	0.76	0.15	1.1	0.11	1.6	0.56	1.2	0.39
CYP2E1														
CYP2J2	1.0	0.56	1.4	0.92	1.1	0.62	1.5	1.5	2.0	1.6	1.9	1.4	1.7	0.95
CYP3A4	65	9.6	91	24	75	13	78	31	75	24	59	30	54	9
CYP3A5	0.63	0.18	0.87	0.22	0.59	0.11	0.75	0.12	0.99	0.23	1.4	0.50	1.3	0.53
CYP3A7														
CYP4A11														
CYP4F2	9.7	0.35	14	2.9	10	0.50	13	0.96	12	0.42	14	0.87	13	0.47

**Protein**	**IN006**						
	**Duodenum**	**Proximal jejunum**	**Mid-jejunum**	**Distal jejunum**	**Proximal ileum**	**Mid-ileum**	**Distal ileum**

	Mean	SD	Mean	SD	Mean	SD	Mean	SD	Mean	SD	Mean	SD	Mean	SD
CYP1A1	NA		ND		ND		ND		ND		ND		ND	
CYP1A2	NA		ND		ND		ND		ND		ND		ND	
CYP1B1	NA		ND		ND		ND		ND		ND		ND	
CYP2A6	NA		ND		ND		ND		ND		ND		ND	
CYP2B6	NA		ND		ND		ND		ND		ND		ND	
CYP2C8	NA		ND		ND		ND		ND		ND		ND	
CYP2C9	NA		2.02	1.7	2.7	2.34	2.1	1.9	2.4	2.2	1.1	1.1	2.0	1.6
CYP2C19	NA		0.92	0.49	0.98	0.26	1.1	0.70	0.20	0.10	0.19	0.14	0.18	0.08
CYP2D6	NA		0.25	0.22	0.44	0.40	0.21	0.23	0.61	0.42	0.70	0.50	0.95	0.68
CYP2E1	NA		ND		ND		ND		ND		ND		ND	
CYP2J2	NA		0.93	0.10	1.1	0.58	1.1	0.34	2.7	1.6	1.9	1.0	1.9	0.56
CYP3A4	NA		44	7.1	57	17	45	16	39	21	30	17	44	10
CYP3A5	NA		0.53	0.53	0.77	0.76	0.62	0.64	0.72	0.53	0.75	0.53	0.80	0.59
CYP3A7	NA		ND		ND		ND		ND		ND		ND	
CYP4A11	NA		ND		ND		ND		ND		ND		ND	
CYP4F2	NA		2.4	0.34	3.7	0.27	2.5	0.53	3.9	0.18	3.3	0.25	3.6	0.32

Protein	IN007

	Duodenum		Proximal jejunum		Mid-jejunum		Distal jejunum		Proximal ileum		Mid-ileum		Distal ileum	
	**Mean**	**SD**	**Mean**	**SD**	**Mean**	**SD**	**Mean**	**SD**	**Mean**	**SD**	**Mean**	**SD**	**Mean**	**SD**
CYP1A1	NA		ND		ND		ND		ND		ND		ND	
CYP1A2	NA		ND		ND		ND		ND		ND		ND	
CYP1B1	NA		ND		ND		ND		ND		ND		ND	
CYP2A6	NA		ND		ND		ND		ND		ND		ND	
CYP2B6	NA		ND		ND		ND		ND		ND		ND	
CYP2C8	NA		ND		ND		ND		ND		ND		ND	
CYP2C9	6.7	1.3	6.6	2.3	6.1	1.4	5.5	2.3	2.6	1.9	2.45	1.9	1.9	1.0
CYP2C19	1.2	0.19	0.98	0.22	0.81	0.20	0.69	0.22	0.22	0.12	0.28	0.20	0.35	0.12
CYP2D6	0.70	0.08	0.80	0.08	0.60	0.09	0.58	0.14	0.30	0.07	0.40	0.23	0.64	0.37
CYP2E1	NA		ND		ND		ND		ND		ND		ND	
CYP2J2	0.51	0.43	0.66	0.46	0.59	0.49	0.75	0.68	0.70	0.59	0.92	0.68	0.70	0.24
CYP3A4	84	16	100	20	85	17	82	34	57	26	64	30	58	6.5
CYP3A5	0.58	0.13	0.56	0.20	0.58	0.07	0.71	0.17	0.48	0.26	0.57	0.51	0.54	0.44
CYP3A7	NA		ND		ND		ND		ND		ND		ND	
CYP4A11	NA		ND		ND		ND		ND		ND		ND	
CYP4F2	4.5	0.22	7.2	0.27	6.2	0.42	6.8	1.2	5.5	0.20	6.0	0.29	3.7	0.21

**Protein**	**IN008**						
	**Duodenum**	**Proximal jejunum**	**Mid-jejunum**	**Distal jejunum**	**Proximal ileum**	**Mid-ileum**	**Distal ileum**

	Mean	SD	Mean	SD	Mean	SD	Mean	SD	Mean	SD	Mean	SD	Mean	SD
CYP1A1	ND		ND		ND		ND		ND		ND		ND	
CYP1A2	ND		ND		ND		ND		ND		ND		ND	
CYP1B1	ND		ND		ND		ND		ND		ND		ND	
CYP2A6	ND		ND		ND		ND		ND		ND		ND	
CYP2B6	ND		ND		ND		ND		ND		ND		ND	
CYP2C8	ND		ND		ND		ND		ND		ND		ND	
CYP2C9	8.1	0.81	14	1.4	6.6	2.4	10	4.6	4.0	1.3	3.5	1.2	3.3	1.1
CYP2C19	2.3	0.72	2.3	0.57	1.1	0.43	1.1	0.45	0.68	0.26	0.79	0.30	0.52	0.24
CYP2D6	0.78	0.04	1.3	0.07	0.74	0.21	1.3	0.42	1.3	0.19	1.6	0.35	2.0	0.38
CYP2E1	ND		ND		ND		ND		ND		ND		ND	
CYP2J2	0.79	0.52	1.2	0.83	1.1	0.57	1.9	1.5	1.4	0.77	1.3	0.76	1.2	0.75
CYP3A4	48	5.0	70	8.6	57	7.2	98	26	46	2.4	40	5.0	40	5.3
CYP3A5	0.69	0.19	0.79	0.18	0.51	0.27	0.88	0.43	0.61	0.27	0.76	0.31	0.80	0.32
CYP3A7	ND		ND		ND		ND		ND		ND		ND	
CYP4A11	ND		ND		ND		ND		ND		ND		ND	
CYP4F2	3.7	0.14	7.2	0.20	5.2	0.40	9.8	2.1	6.0	0.38	5.7	0.77	5.1	0.46

**Protein**	**IN009**

	Duodenum		Proximal jejunum		Mid-jejunum		Distal jejunum		Proximal ileum		Mid-ileum		Distal ileum	
	**Mean**	**SD**	**Mean**	**SD**	**Mean**	**SD**	**Mean**	**SD**	**Mean**	**SD**	**Mean**	**SD**	**Mean**	**SD**
CYP1A1	ND		ND		ND		ND		ND		ND		ND	
CYP1A2	ND		ND		ND		ND		ND		ND		ND	
CYP1B1	ND		ND		ND		ND		ND		ND		ND	
CYP2A6	ND		ND		ND		ND		ND		ND		ND	
CYP2B6	ND		ND		ND		ND		ND		ND		ND	
CYP2C8	ND		ND		ND		ND		ND		ND		ND	
CYP2C9	3.2	0.94	5.5	3.6	5.6	2.9	3.1	1.3	2.1	1.8	2.7	1.4	1.1	0.70
CYP2C19	0.39	0.14	0.23	0.20	0.18	0.13	0.08	0.07	0.25	0.28	0.19	0.13	0.03	0.03
CYP2D6	0.06	0.05	0.13	0.06	0.20	0.07	0.13	0.13	0.02	0.03	0.23	0.09	0.12	0.04
CYP2E1	ND		ND		ND		ND		ND		ND		ND	
CYP2J2	0.39	0.39	0.63	0.67	0.50	0.53	0.42	0.46	0.57	0.56	1.5	0.84	0.54	0.36
CYP3A4	13	4.3	16	7.8	15	4.8	8.9	4.8	9.9	3.7	17	3.2	12	4.0
CYP3A5	0.50	0.09	0.52	0.24	0.56	0.11	0.39	0.08	0.29	0.26	0.82	0.45	0.51	0.29
CYP3A7	ND		ND		ND		ND		ND		ND		ND	
CYP4A11	ND		ND		ND		ND		ND		ND		ND	
CYP4F2	1.5	0.27	2.4	0.51	2.4	0.50	1.7	0.41	1.8	0.25	3.5	0.51	2.1	0.20

Table S8. CYP450 allele frequencies among 9 donors.

Gene	Allelic variant	Frequency % (n)	Protein expression (pmol/mg protein)
CYP2C9	*CYP2C9*1*	61% (11)	5.6 ± 2.9
	*CYP2C9*2*	28% (5)	5.6 ± 2.1
	*CYP2C9*3*	11% (2)	5.7 ± 0.47
CYP2C19	*CYP2C19*1*	83% (15)	1.0 ± 0.63
	*CYP2C19*2*	0%	
	*CYP2C19*3*	0%	
	*CYP2C19*17*	17% (3)	0.98 ± 0.84
CYP2D6	*CYP2D6*1*	22% (4)	0.68 ± 0.49
	*CYP2D6*2*	28% (5)	0.78 ± 0.33
	*CYP2D6*3*	6% (1)	0.30
	*CYP2D6*4*	33% (6)	0.50 ± 0.44
	*CYP2D6*5 (del)*	0%	
	*CYP2D6*6*	0%	
	*CYP2D6*10*	0%	
	*CYP2D6*41*	11% (2)	0.39 ± 0.44
	*CYP2D6*N*	0%	
CYP2J2	*CYP2J2*1*	94% (17)	0.95 ± 0.45
	*CYP2J2*6*	0%	
	*CYP2J2*7*	6% (1)	1.7
CYP3A4	*CYP3A4*1*	94% (17)	50.3 ± 27.2
	*CYP3A4*22*	6% (1)	13.2
CYP3A5	*CYP3A5*1*	0%	
	*CYP3A5*3*	100% (18)	0.65 ± 0.11
	*CYP3A5*6*	0%	

Table S9. Protein expression average in jejunum section according to sex, age and body mass index. Protein amounts are expressed in pmol/mg prot.

**CYP450/Sex**	**Men (N=5)**	**Women (N=4)**	***P* value**
CYP2C9	6.7 ± 2.9	4.2 ± 1.9	0.2857
CYP2C19	0.95 ± 0.73	1.1 ± 0.58	0.7143
CYP2D6	0.68 ± 0.35	0.36 ± 0.47	0.2857
CYP2J2	0.78 ± 0.35	1.2 ± 0.52	0.5556
CYP3A4	55 ± 29	45 ± 28	0.8254
CYP3A5	0.62 ± 0.15	0.68 ± 0.04	0.5079
CYP4F2	6.6 ± 2.9	5.4 ± 4.7	0.6825

**CYP450/Age**	**0-49 (N=4)**	**>50 (N=5)**	***P* value**
CYP2C9	6.9 ± 3.6	4.5 ± 1.3	0.2857
CYP2C19	1.5 ± 0.37	0.66 ± 0.56	0.0635
CYP2D6	0.53 ± 0.48	0.55 ± 0.42	> 0.999
CYP2J2	1.2 ± 0.46	0.73 ± 0.34	0.0635
CYP3A4	52 ± 17	49 ± 36	0.8730
CYP3A5	0.71 ± 0.07	0.60 ± 0.12	0.2143
CYP4F2	4.9 ± 2.3	7.0 ± 4.4	0.5238

**CYP450/BMI**	**<30 (N=5)**	**>30 (N=4)**	***P* value**
CYP2C9	5.6 ± 3.2	5.6 ± 2.4	> 0.999
CYP2C19	1.2 ± 0.52	0.81 ± 0.77	0.3968
CYP2D6	0.65 ± 0.48	0.40 ± 0.33	0.3968
CYP2J2	1.2 ± 0.41	0.59 ± 0.09	0.0635
CYP3A4	57 ± 20	42 ± 35	0.5238
CYP3A5	0.64 ± 0.11	0.65 ± 0.13	0.9524
CYP4F2	7.2 ± 4.3	4.6 ± 2.2	0.3968

Table S10. Proteospecific peptides sequences, MRM transitions (precursor to products transitions), and collision energy (CE) used for quantification. The labeling of lysine or arginine terminal is indicated in the internal standard sequence by K^ or R^.

Protein	Proteotypic peptide	Mass	Precursor (m/z)	z	Product (m/z)	Ion/z	CE (eV)	Product (m/z)	Ion/z	CE (eV)	Product (m/z)	Ion/z	CE (eV)
CYP1A1	LAQNGLK	742.9	372.2	2+	431.2	y4/1+	14.9	559.3	y5/1+	12.7	630.3	y6/1+	10.3
	LAQNGLK^	750.8	376.2	2+	439.2	y4/1+	14.9	567.4	y5/1+	12.7	638.3	y6/1+	10.3
	YLPNPSLNAFK	1263.5	632.3	2+	494.2	y9/2+	15.9	776.4	y7/1+	24.3	987.5	y9/1+	17.4
	YLPNPSLNAFK^	1271.4	636.3	2+	498.3	y9/2+	15.9	784.5	y7/1+	24.3	995.5	y9/1+	17.4
CYP1A2	DTTLNGFYIPK	1268.4	634.8	2+	244.1	y2/1+	16.2	724.4	y6/1+	19.5	838.5	y7/1+	16.2
	DTTLNGFYIPK^	1276.4	638.8	2+	252.2	y2/1+	16.2	732.4	y6/1+	19.5	846.5	y7/1+	16.2
	YLPNPALQR	1071.2	536.3	2+	398.3	y7/2+	14.1	584.4	y5/1+	17.2	795.5	y7/1+	17.0
	YLPNPALQR^	1081.2	541.3	2+	403.3	y7/2+	14.1	594.4	y5/1+	17.2	805.5	y7/1+	17.0
CYP1B1	ELVALLVR	912.1	456.8	2+	387.2	y3/1+	12.2	500.3	y4/1+	13.2	571.4	y5/1+	16.1
	ELVALLVR^	922.1	461.8	2+	397.3	y3/1+	12.2	510.3	y4/1+	13.2	581.4	y5/1+	16.1
	YGDVFQIR	997.1	499.3	2+	336.1	b3/1+	18.0	563.4	y4/1+	11.2	662.4	y5/1+	16.1
	YGDVFQIR^	1007.0	504.3	2+	336.0	b3/1+	18.0	573.3	y4/1+	11.2	672.4	y5/1+	16.1
CYP2B6	GYGVIFANGNR	1167.3	584.3	2+	531.2	y5/1+	21.1	678.3	y6/1+	16.6	791.4	y7/1+	15.4
	GYGVIFANGNR^	1177.2	589.3	2+	541.2	y5/1+	21.1	688.4	y6/1+	16.6	801.4	y7/1+	15.4
	NLQEINAYIGHSVEK	1714.9	572.3	3+	656.3	y6/1+	14.7	744.5	y13/2+	14.1	769.4	y7/1+	23.8
	NLQEINAYIGHSVEK^	1722.8	575.0	3+	664.3	y6/1+	14.7	748.3	y13/2+	14.1	777.4	y7/1+	23.8
CYP2C8	NLNTTAVTK	961.1	481.3	2+	519.3	y5/1+	18.0	620.4	y6/1+	20.4	734.3	y7/1+	10.3
	NLNTTAVTK^	969.0	485.3	2+	527.2	y5/1+	18.0	628.4	y6/1+	20.4	742.4	y7/1+	10.3
	VQEEIDHVIGR	1294.4	432.2	3+	444.2	y4/1+	17.2	534.4	y9/2+	10.3	581.3	y5/1+	21.0
	VQEEIDHVIGR^	1304.4	435.6	3+	454.3	y4/1+	17.2	539.3	y9/2+	10.3	591.2	y5/1+	21.0
CYP2C9	GIFPLAER	902.1	451.8	2+	293.2	y5/2+	16.9	366.6	y6/2+	11.7	585.4	y5/1+	13.5
	GIFPLAER^	912.0	456.8	2+	298.1	y5/2+	16.9	371.7	y6/2+	11.7	595.3	y5/1+	13.5
	SLVDPK	657.8	329.7	2+	201.1	b2/1+	11.5	244.1	y2/1+	10.3	458.2	y4/1+	10.3
	SLVDPK^	665.7	333.7	2+	201.2	b2/1+	11.5	252.2	y2/1+	10.3	466.2	y4/1+	10.3
CYP2C19	GHFPLAER	926.0	463.8	2+	342.2	b3/1+	10.3	585.3	y5/1+	13.7	732.4	y6/1+	14.6
	GHFPLAER^	936.0	468.8	2+	342.0	b3/1+	10.3	595.3	y5/1+	13.7	742.4	y6/1+	14.6
	GTTILTSLTSVLHDNK	1699.9	567.3	3+	608.0	y11/2+	15.8	664.5	y12/2+	18.1	771.5	y14/2+	10.3
	GTTILTSLTSVLHDNK^	1707.9	570.0	3+	611.8	y11/2+	15.8	668.6	y12/2+	18.1	775.6	y14/2+	10.3
CYP2D6	DIEVQGFR	963.1	482.2	2+	507.3	y4/1+	14.5	606.3	y5/1+	16.0	735.4	y6/1+	13.4
	DIEVQGFR^	973.0	487.3	2+	517.2	y4/1+	14.5	616.2	y5/1+	16.0	745.4	y6/1+	13.4
	SQGVFLAR	877.0	439.2	2+	506.3	y4/1+	12.1	605.4	y5/1+	17.3	662.5	y6/1+	13.8
	SQGVFLAR^	886.9	444.3	2+	516.3	y4/1+	12.1	615.3	y5/1+	17.3	672.4	y6/1+	13.8
CYP2E1	FITLVPSNLPHEATR	1695.0	565.9	3+	561.3	y10/2+	15.0	710.3	y6/1+	22.8	718.1	y13/2+	12.3
	FITLVPSNLPHEATR^	1704.9	569.1	3+	566.2	y10/2+	15.0	720.3	y6/1+	22.8	722.9	y13/2+	12.3
	LHEEIDR	911.0	456.3	2+	251.1	b2/1+	17.6	399.6	y6/2+	17.2	532.2	y4/1+	19.5
	LHEEIDR^	920.9	461.3	2+	251.2	b2/1+	17.6	404.5	y6/2+	17.2	542.4	y4/1+	19.5
CYP2J2	DFIDAYLK	984.1	492.8	2+	263.0	b2/1+	12.4	609.3	y5/1+	13.9	722.4	y6/1+	13.2
	DFIDAYLK^	992.1	496.8	2+	263.0	b2/1+	12.4	617.3	y5/1+	13.9	730.4	y6/1+	13.2
	VQAEIDR	829.9	415.7	2+	228.1	b2/1+	12.5	532.2	y4/1+	14.8	603.3	y5/1+	14.2
	VQAEIDR^	839.8	420.7	2+	228.1	b2/1+	12.5	542.3	y4/1+	14.8	613.3	y5/1+	14.2

CYP3A4	EVTNFLR	878.0	439.7	2+	288.2	y2/1+	17.0	435.2	y3/1+	16.5	549.3	y4/1+	12.9
	EVTNFLR^	887.9	444.7	2+	298.3	y2/1+	17.0	445.4	y3/1+	16.5			
	LQEEIDAVLPNK	1368.6	684.9	2+	358.1	y3/1+	20.6	471.2	y4/1+	21.0	1127.5	y10/1+	18.4
	LQEEIDAVLPNK^	1376.5	688.9	2+	366.2	y3/1+	20.6	479.2	y4/1+	21.0	1135.6	y10/1+	18.4
CYP3A5	DVEINGVFIPK	1230.4	615.8	2+	774.4	y7/1+	18.7	887.5	y8/1+	17.5	987.5	b9/1+	13.1
	DVEINGVFIPK^	1238.4	619.9	2+	782.5	y7/1+	18.7	895.6	y8/1+	17.5	987.5	b9/1+	13.1
	LFPVAIR	815.0	408.2	2+	278.1	y5/2+	10.3	458.2	y4/1+	21.1	555.4	y5/1+	13.6
	LFPVAIR^	825.0	413.3	2+	283.0	y5/2+	10.3	468.2	y4/1+	21.1	565.4	y5/1+	13.6
CYP3A7	FNPLDPFVLSIK	1389.7	695.4	2+	564.8	y10/2+	18.2	918.4	y8/1+	25.9	1128.7	y10/1+	15.5
	FNPLDPFVLSIK^	1397.6	699.4	2+	569.0	y10/2+	18.2	926.6	y8/1+	25.9	1136.7	y10/1+	15.5
	VISFLTK	807.0	404.2	2+	248.1	y2/1+	17.4	595.2	y5/1+	10.3	708.5	y6/1+	14.8
	VISFLTK^	814.9	408.3	2+	256.2	y2/1+	17.4	603.3	y5/1+	10.3	716.5	y6/1+	14.8
CYP4F2	HVTQDIVLPDGR	1349.5	450.6	3+	557.2	y5/1+	12.7	581.2	b5/1+	13.4	694.4	b6/1+	10.3
	HVTQDIVLPDGR^	1359.4	453.9	3+	567.3	y5/1+	12.7	581.2	b5/1+	13.4	694.3	b6/1+	10.3
	SVINASAAIAPK	1141.3	571.3	2+	478.4	y10/2+	14.1	657.4	y7/1+	16.9	842.5	y9/1+	17.7
	SVINASAAIAPK^	1149.3	575.3	2+	482.3	y10/2+	14.1	665.3	y7/1+	16.9	850.5	y9/1+	17.7

Text S1: CYP2A6 and CYP4A11 quantification method validation

Two proteotypic peptides were selected for CYP2A6 (GTGGANIDPTFFLSR and DPSFFSNPQDFNPQHFLNEK) and CYP4A11 (VATALTLLR and NAFHQNDTIYSLTSAGR). Stable isotope-labelled internal standards, with ^{13}C labeled lysine (K) or ^{15}N labeled arginine (R) in C-terminal, were synthetized for all peptides. The amino acid sequences of the proteotypic peptides and their internal standard are reported in Table S1.

CYP2A6 and CYP4A11 quantification method was validated according to the current U.S. FDA guidelines for bioanalytical methods as no guidelines for proteomics assay are yet developed [1]. The assay was linear over a range of 0.1 to 15 nM and a linear regression (1/X) produced the best fit for the concentration-detector relationship. The correlation coefficients (r^2) were greater than 0.9947, the lower limit of quantification (LLOQ) precision was better than 19.7% and the LLOQ accuracy was between 80.7-118.8%. The intra-day (n=6) and the inter-day (n=18) precision and accuracy were assessed by replicate analyses of quality control (QC) samples at three concentrations (low, mid, high) within an analytical run or within three independent analytical runs, respectively. For these QC samples, the precision was better than 11.4% and the accuracy was in the 88.0-112.1% range. The precision and accuracy results are shown in Table S2. Matrix effect and autosampler stability were evaluated by back-calculating QC concentrations against the calibration curve. Matrix effect was evaluated in six lots of commercially pooled HIM and digested HIM were fortified with mid QC, high QC or ACN:H_2O solution (40:60, v/v)) in triplicate. Autosampler stability was assessed by keeping low, mid and high QC samples in the autosampler for 98 h at 15°C. Percent nominal was between 80.3-119.2% and 88.0-109.2% for matrix effect (Table S3) and autosampler stability (Table S4), respectively.

For each CYP450, protein quantification was measured with two proteotypic peptide. Final CYP450 protein amount was calculated as the average of these two measured peptides. Correlation analyses between the two proteotypic peptides were assessed using pooled and individual human liver microsomes (n=16). Correlation analyses were evaluated using the Spearman rank test. Strong correlations were observed (r 0.679) and y-intercepts were -2.764 (CYP2A6) and 4.099 (CYP4A11) (Table S5). CYP2A6 and 4A11 quantification methods were also evaluated in both commercially and in-house human intestinal microsomes, but these isoenzymes were not measured in any intestinal microsome (Table S6).

[1] U.D.o.H.a.H.S. FDA Guidance, Food and Drug Administration, Center for Drug Evaluation and Research (CDER), Center for Veterinary Medicine (CVM), Guidance for Industry. Bioanalytical Method Validation, 2001.
